# A recombinant subunit vaccine candidate produced in plants elicits neutralizing antibodies against SARS-CoV-2 variants in macaques

**DOI:** 10.3389/fpls.2022.901978

**Published:** 2022-09-28

**Authors:** Narach Khorattanakulchai, Kanjana Srisutthisamphan, Balamurugan Shanmugaraj, Suwimon Manopwisedjaroen, Kaewta Rattanapisit, Chalisa Panapitakkul, Taratorn Kemthong, Nutchanat Suttisan, Suchinda Malaivijitnond, Arunee Thitithanyanont, Anan Jongkaewwattana, Waranyoo Phoolcharoen

**Affiliations:** ^1^Center of Excellence in Plant-produced Pharmaceuticals, Chulalongkorn University, Bangkok, Thailand; ^2^Department of Pharmacognosy and Pharmaceutical Botany, Faculty of Pharmaceutical Sciences, Chulalongkorn University, Bangkok, Thailand; ^3^Virology and Cell Technology Laboratory, National Center for Genetic Engineering and Biotechnology (BIOTEC), National Science and Technology Development Agency, Pathum Thani, Thailand; ^4^Baiya Phytopharm Co., Ltd., Bangkok, Thailand; ^5^Department of Microbiology, Faculty of Science, Mahidol University, Bangkok, Thailand; ^6^National Primate Research Center of Thailand-Chulalongkorn University, Saraburi, Thailand

**Keywords:** COVID-19, SARS-CoV-2, Baiya SARS-CoV-2 Vax 1, plant-produced recombinant protein, subunit vaccine, immunogenicity

## Abstract

Since the outbreak of the coronavirus disease (COVID) pandemic in 2019, the development of effective vaccines to combat the infection has been accelerated. With the recent emergence of highly transmissible severe acute respiratory syndrome coronavirus 2 (SARS-CoV-2) variants of concern (VOC), there are concerns regarding the immune escape from vaccine-induced immunity. Hence an effective vaccine against VOC with a potent immune response is required. Our previous study confirmed that the two doses of the plant-produced receptor-binding domain (RBD) of SARS-CoV-2 fused with the Fc region of human IgG1, namely Baiya SARS-CoV-2 Vax 1, showed high immunogenicity in mice and monkeys. Here, we aimed to evaluate the immunogenicity of a three-dose intramuscular injection of Baiya SARS-CoV-2 Vax 1 on days 0, 21, and 133 in cynomolgus monkeys. At 14 days after immunization, blood samples were collected to determine RBD-specific antibody titer, neutralizing antibody, and pseudovirus neutralizing antibody titers. Immunized monkeys developed significantly high levels of antigen-specific antibodies against SARS-CoV-2 compared to the control group. Interestingly, the sera collected from immunized monkeys also showed a neutralizing antibody response against the SARS-CoV-2 VOCs; Alpha, Beta, Gamma, Delta, and Omicron. These findings demonstrate that a three-dose regimen of Baiya SARS-CoV-2 Vax 1 vaccine elicits neutralizing immune response against SARS-CoV-2 variants.

## Introduction

Severe acute respiratory syndrome coronavirus 2 (SARS-CoV-2) was first reported in late December 2019 in Wuhan, China (Huang et al., 2020; Li et al., 2020). This virus is responsible for an acute respiratory condition known as coronavirus disease (COVID). Within weeks of its outbreak, World Health Organization (WHO) announced COVID-19 as a pandemic disease (World Health Organization, 2020b). According to the data as of July 15, 2022, over 557 million cases have been confirmed and 6 million deaths have been reported, (World Health Organization, 2020a). Earlier reports suggested that SARS-CoV-2, similar to SARS-CoV (Lu et al., 2020), binds to human angiotensin-converting enzyme 2 (ACE2) receptor *via.*, spike or surface (S) glycoprotein for infection (Hoffmann et al., 2020; Shang et al., 2020; Zhao et al., 2020). S protein plays an important role in host cell attachment, cleaved into S1 and S2 subunits by the host proteases during the infection process. In particular, the receptor-binding domain (RBD), located in the S1 subunit of SARS-CoV-2, recognizes ACE2 in the host cell and plays a crucial role in host cell entry. Therefore, the S, S1, or RBD proteins are the primary targets for neutralizing antibodies and therapeutic vaccine development (Krammer, 2020; Shanmugaraj et al., 2020b; Smith et al., 2020; Wang et al., 2020a).

Since its outbreak, several pharmaceutical companies, academic laboratories, and research institutions attempt to develop vaccines against SARS-CoV-2 (World Health Organization, 2021a). Recently, WHO has approved a few vaccines under emergency use listing (EUL), including those produced by Pfizer/BioNTech (USA), Oxford/AstraZeneca-SK Bio (UK), Janssen (USA), Moderna (USA), Sinopharm/BIBP (China), Sinovac (China), Bharat Biotech (India), and Novavax (USA) (World Health Organization, 2022). The new variants of SARS-CoV-2 have been predominately emerging worldwide (Abdool Karim and De Oliveira, 2021; Singh et al., 2021a), that exhibit high virulence, fast transmission, and reduced susceptibility to neutralizing antibodies (World Health Organization, 2021b). Data as of July 17, 2022, Alpha (B.1.1.7, United Kingdom), Beta (B.1.351, South Africa), Gamma (P.1, Brazil), Delta (B.1.617.2, India), and Omicron (B.1.1.529) are VOCs containing multiple mutations on the RBD, which could reduce vaccine efficacy (Abdool Karim and De Oliveira, 2021; Hacisuleyman et al., 2021; Kustin et al., 2021; Zhou et al., 2021). BNT162b2 (Pfizer)-elicited antibodies showed a 2.6-fold and 8.8-fold reduction in neutralizing Alpha and Beta compared to Wuhan (Bates et al., 2021), whereas it showed 3-fold and 16-fold decrease in the neutralization titers against the Delta and the Beta variants, respectively, compared to the Alpha variant were reported. With two doses of AstraZeneca vaccine, 5-fold and 9-fold reductions in neutralization titers against the Delta and the Beta variants relative to the Alpha variant were reported. Both Pfizer and AstraZeneca vaccines showed neutralizing responses against the Delta variant only after the second dose (Planas et al., 2021). Similarly, mRNA-1273 elicited antibodies showed a 2.3-fold reduction in neutralizing alpha relative to the Wuhan strain (Garcia-Beltran et al., 2021). Hence urgent research is required to fill the knowledge gaps on SARS-CoV-2 and its variants for effective vaccine design and treatment to combat its infection. In addition, the efficacy of available vaccines and other potential candidates against VOCs should be investigated.

Subunit vaccines based on synthetic peptides or recombinant proteins have been developed for SARS-CoV and MERS-CoV and are shown to be effective in animal models (Wang et al., 2020b). Similarly, subunit vaccines against SARS-CoV-2 developed by Novavax (full-length S protein), Anhui Zhifei Longcom-Chinese Academy of Sciences (dimeric RBD-based), and Kentucky Bioprocessing Inc. (RBD-based) showed promising results in clinical trials (Keech et al., 2020; Pollet et al., 2021; Yang et al., 2021b). Nevertheless, the cost-effective platform for producing recombinant subunit vaccines might reduce the overall vaccine cost, which, in turn, reduces the financial burden and improve global vaccine accessibility. Recently, the plant expression system is considered an alternative to conventional platforms to produce recombinant proteins, enzymes, therapeutic antigens, antimicrobial peptides, diagnostic/research reagents, and monoclonal antibodies, especially during emergency situations (Capell et al., 2020; Kumar et al., 2021; Lobato Gomez et al., 2021; Singh et al., 2021b). Plant platform offers many advantages such as rapid scale-up, cost-effective, and low human pathogen contamination (Gleba et al., 2005; Rosales-Mendoza et al., 2017; Sohrab et al., 2017; Zhang et al., 2017; Shanmugaraj et al., 2020a; Leblanc et al., 2021). Recently, our group reported that the plant-produced recombinant SARS-CoV-2 RBD fused with fragment crystallizable (Fc) region of human IgG1 (RBD-Fc) adjuvanted with alum, namely Baiya SARS-CoV-2 Vax 1, elicited potent neutralizing antibodies in both mice and cynomolgus monkeys (Siriwattananon et al., 2021; Shanmugaraj et al., 2022). The Fc fragment has been tagged to the RBD owing to its advantages such as easy, rapid purification, longer half-life, and also their polymeric nature provides an additional antigen depot effect (Sun et al., 2021). Fc-fused protein vaccines against SARS-CoV and influenza have also been reported (Du et al., 2007; Li et al., 2013). The current study aimed to expand our horizon of the previous knowledge on Baiya SARS-CoV-2 Vax 1, by investigating the immune response of low doses of our vaccine with a three-dose regimen in cynomolgus monkeys. Further, the ability of antibodies elicited by Baiya SARS-CoV-2 Vax 1 to neutralize SARS-CoV-2 variants was also elucidated.

## Materials and methods

### Preparation of recombinant RBD-Fc protein

The cloning and expression of RBD-Fc fusion protein have been provided in detail in our previous report (Siriwattananon et al., 2021). Briefly, the RBD-Fc fusion construct was constructed from the RBD of SARS-CoV-2 (YP_009724390.1, F318-C617) containing 3XGGGGS linker fused with fragment crystallizable (Fc) region of human IgG1 (4CDH_A, P35-K255) was cloned into the plant expression vector pBYR2eK2Md (pBY2eK) (Chen et al., 2011; Diamos and Mason, 2018). Then the expression vector harboring the RBD-Fc fusion gene was transformed into *Agrobacterium tumefaciens* GV3101. The recombinant *Agrobacterium* was cultured for transient protein expression in *N. benthamiana* by agroinfiltration. After 4-days post-infiltration, the infiltrated leaves were harvested and protein was extracted. The crude extract was purified by protein A affinity chromatography (GE Healthcare, USA) and sterile filtered by a 0.22 μm syringe filter (Merck, USA). The purified RBD-Fc was analyzed by sodium dodecyl-sulfate polyacrylamide gel electrophoresis (SDS-PAGE) and confirmed by western blotting with goat anti-human IgG-HRP (Southern Biotech, USA), and anti-SARS-CoV-2 RBD-conjugated HRP (Sino Biological, Beijing, China).

### Vaccine formulation

Baiya SARS-CoV-2 Vax 1 vaccine is a plant-produced SARS-CoV-2 RBD-Fc fusion protein with alum as an adjuvant. Baiya SARS-CoV-2 Vax 1 vaccine at a dose of 10 μg protein and 0.5 mg alum was used in this study. The control was phosphate-buffered saline (PBS) containing alum without plant-produced RBD-Fc. A total volume of 0.5 ml was injected intramuscularly into the quadriceps femoris muscle of animals.

### Immunization in cynomolgus monkeys

The study was performed in the National Primate Research Center of Thailand-Chulalongkorn University (NPRCT-CU; AAALAC International Accredited facility). Ten female cynomolgus monkeys (*Macaca fascicularis*) originated from Thailand, aged 3–5 years and body weight between 2.4 and 3.1 kg, were supplied by the NPRCT-CU breeding facility. They were specific pathogen-free (SPF) animals that were free from tuberculosis, B virus, SRV, SIV, STLV, and SARS-CoV-2. The monkeys were kept in the individual cage in the experimental room of biosafety level 1 at a temperature of 25 ± 2 °C, relative humidity of 60 ± 10%, and illumination cycle of 12 h light: 12 h dark (lights on at 06.00 a.m.). The environmental condition in the experimental room was recorded two times a day (morning and afternoon). The measurement of temperature and relative humidity was recorded in accordance with SOP Facility Management (SOP PM07) of NPRCT-CU. The monkeys were fed with standard monkey chow (Perfect Companion Group Co., Ltd., Thailand) in the morning (09.00–10.00 a.m.) and fresh fruits and vegetables in the afternoon (14.30–15.30 p.m.) and drinking water *ad libitum*. However, the feeding time in the morning on the day of vaccine administration and blood collection was from 09.30 to 11.30 a.m. after the animals were fully recovered from the anesthesia. In general, the animals were not anesthetized in this study, except during vaccine administrations and blood collections which were needed to avoid discomfort/pain/distress that may affect the accuracy of the observation. The animal's health was monitored daily by the veterinarian or veterinary technician. The abnormal signs of the health and wellbeing of the animals were diagnosed by the veterinarian based on the PM06/WI01: Guide on Clinical Signs and Symptom in Cynomolgus Monkeys of the NPRCT-CU.

Animals were randomly divided into two groups (*n* = 5); Baiya SARS-CoV-2 Vax 1 vaccine and control group. Monkeys were intramuscularly injected with 0.5 mL of either vaccine or alum alone on days 0, 21, and 133. The blood samples were collected on day 0 (before the first injection) and day 133 (before the third injection), and 14 days after each immunization on days 14, 35, 133, and 147, to assess the antigen-specific antibody titer, live virus neutralizing antibody, and for pseudovirus neutralization antibody titers. Monkeys were anesthetized in the morning for health monitoring and returned to their home cages of social housing at the breeding facility after the experiment was completed. The animal use and experimental procedures have been approved by the NPRCT-CU Animal Care and Use Committee (Protocol review no. 2075015).

### Evaluation of RBD-specific antibody titer by enzyme-linked immunosorbent assay (ELISA)

SARS-CoV-2 spike protein RBD (100 ng/well; Cat. No. Z03479; GenScript, Piscataway, NJ, USA) was coated on a high binding 96-well plate (Greiner bio-one, Frickenhausen, Germany) and incubated for overnight at 4°C. Subsequently, the wells were blocked with 5% skim milk powder (BD Difco, Sparks, MD, USA) in 1xPBS pH 7.4 for 2 h at 37°C. Then, the monkey sera were added by starting at 1:100 with 2-fold serial dilution in 1xPBS. The diluted sera were loaded in each well as duplicates and incubated for 2 h at 37°C. After washing, 1:2,000 of goat anti-monkey IgG–HRP conjugate (Abcam, Cambridge, UK) in 1xPBS was added and incubated for 2 h at 37°C. TMB substrate (Promega, Madison, WI, USA) was added to the plates, and then the reactions were stopped by adding 1M H_2_SO_4_. The absorbance at 450 nm (A_450_) was measured by SpectraMax^®^ M3 Microplate Reader (Molecular Devices, San Jose, CA, USA). The plates were washed by 1xPBS with 0.05% Tween 20 (PBST) three times between each step. The endpoint titers were determined as the highest dilution of immunized sera, which had A_450_ more than the cut-off value calculated from A_450_ of pre-immunized sera at 1:100 dilution (Frey et al., 1998). The data were plotted as a geometric mean titer (GMT) with a ± 95% confidence interval (CI) by GraphPad Prism software version 9.0 (GraphPad Software, Inc., San Diego, CA, USA). Statistical significance was calculated by two-way analysis of variance (ANOVA) Dunnett's test compared with sera on day 0. The *p* < 0.05 was considered statistically significant.

### Microneutralization assay

Microneutralization assay was performed in 96-well microplates containing confluent Vero E6 cell line and the live ancestral strain SARS-CoV-2 virus (SARS-CoV-2/human/THA/LJ07_P3/2020) isolated from a patient with COVID-19. The experiment was conducted in a certified biosafety level (BSL) 3 facility of the Microbiology Department, Faculty of Science, Mahidol University, Thailand, as previously described (Siriwattananon et al., 2021).

Briefly, immunized monkey sera and the convalescent serum from patients with COVID-19 (positive control) were heat-inactivated at 56°C for 30 min. Two-fold serially diluted sera were mixed with 100 of 50% tissue culture infective dose (TCID_50_) of SARS-CoV-2 in DMEM (Dulbecco's Modified Eagle Medium) at 37°C for 1 h. Virus control and cell control wells were included in all plates. Then, the mixture was applied to a Vero cell monolayer and incubated at 37°C for 2 days. Subsequently, the cells were washed once with 1xPBS, fixed, and permeabilized with chilled 1:1 methanol/acetone fixative solution at 4°C for 20 min. After washing three times with 1xPBST, the plates were blocked with 2% BSA at room temperature (RT) for 1 h. Viral infection was then assessed using 1:5,000 of SARS-CoV/SARS-CoV-2 nucleocapsid (N) monoclonal antibody (Sino Biological, Beijing, China) as a primary antibody and incubated at 37°C for 1 h followed by adding 1:2,000 of HRP-conjugated goat anti-rabbit polyclonal antibodies (Dako, Glostrup, Denmark) in 1xPBS as a secondary antibody and incubated at 37°C for 1 h. The KPL SureBlue^TM^ TMB substrate (SeraCare, Gaithersburg, MD, USA) was added, and the reaction was stopped by 1N HCl. The absorbance was measured using a Sunrise^TM^ microplate reader (Tecan, Männedorf, Switzerland). The differences of A_450_ of samples were compared with the 50% of the cut point, which was calculated as previously described (Seephetdee et al., 2021). Each sample was carried out in duplicates. The data were presented as GMT ± 95% CI by GraphPad Prism software version 9.0. Statistical significance was calculated by two-way analysis of variance (ANOVA) Dunnett's test compared with sera on day 0. The *p* < 0.05 was considered statistically significant.

### Pseudovirus neutralization assay

Lentiviral pseudoviruses bearing CoV spike were constructed as previously described (Hyseni et al., 2020) with minor modifications. Briefly, the combination of plasmids including the lentivirus backbone expressing a firefly luciferase reporter gene (pCSFLW, kindly provided by Dr. Nigel Temperton), the expression plasmid expressing HIV-1 structural/regulatory proteins (pCMVΔR8.91), and pCAGGS expressing the codon-optimized spike gene (Wuhan, Alpha, Beta, Gamma, Delta, and Omicron BA.1 and BA.2) was used to generate pseudoviruses. Unless otherwise indicated, HEK293T/17 producer cells were seeded in 6-well plates at 7.5 × 10^5^/well 24 h before being transfected with the following plasmids: 600 ng pCMVΔR8.91, 600 ng pCSFLW, and 500 ng of pCAGGS-Spike, in OptiMEM (Gibco) with 10 μL of polyethylenimine (PEI). Transfected cells were incubated at 37°C, with 5% CO_2_. At 12 h after transfection, cells were washed and cultured in DMEM-10%. Pooled harvests of supernatants containing pseudoviruses were taken at 72 h post-transfection, centrifuged at 1,500 × *g* for 10 min at 4°C to remove cellular debris, aliquoted, and stored at -80°C.

To titrate pseudoviruses, HEK 293T/17-ACE2 cells were first transfected with the expression plasmid encoding for human TMPRSS2 using FuGENE HD (Promega, Madison, WI, USA) according to the manufacturer's instructions. At 24 h after transfection, the supernatant was replaced by DMEM containing 10% FBS and subsequently used as pseudovirus target cells. Supernatants containing pseudoviruses were serially 2-fold diluted in a DMEM medium in 96-well, flat-bottomed culture plates, and TMPRSS2-expressing HEK 293T/17-ACE2 target cells (1x10^4^ cells/well) were added to each well. After 72 h, the luminescence of cell cultures (in Relative Luminescence Units or RLUs) was evaluated by luminometry (Synergy Plate Reader) using the Bright-Glo assay system (Promega, Madison, WI, USA).

To measure the neutralizing activity of the serum samples, a 2-fold serial dilution of heat-inactivated sera was prepared, starting from 1:40, in a culture medium (DMEM high glucose without FBS). The sera were mixed with pseudoviruses displaying the CoV spike of interest in a 1:1 vol/vol ratio in a 96-well culture plate. The pseudovirus input used was normalized to 1 × 10^5^ RLU/well. The serum–pseudovirus mixture was then incubated for 1 h at 37 °C. Subsequently, cell suspensions of HEK293T-ACE-2 pre-transfected with pCAGGS expressing human TMPRSS2 (2 × 10^4^ cell/ml) were mixed with the serum–pseudovirus mixture seeded into each well of CulturPlate™ Microplates (PerkinElmer). The plates were incubated at 37 °C for 48 h, and the neutralizing antibodies were determined based on luciferase activity as previously described (Ferrara and Temperton, 2018). The data were presented as GMT ± 95% CI by GraphPad Prism software version 9.0. Statistical significance was calculated by two-way analysis of variance (ANOVA) Dunnett's test compared with sera on day 14. The *p* < 0.05 was considered statistically significant.

## Results

### Expression of RBD-Fc fusion protein in *N. benthamiana*

**Figure 1:**
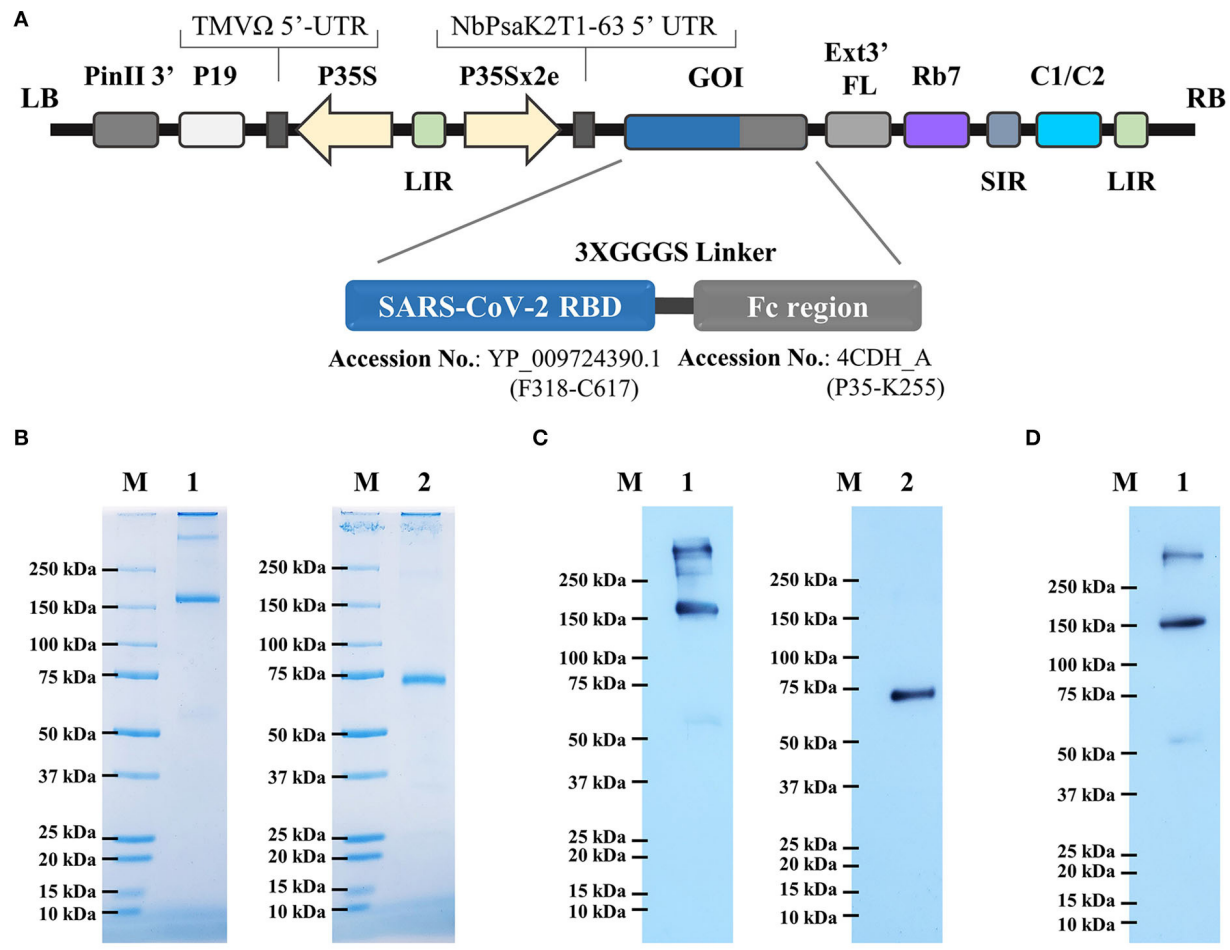
**(A)** Schematic representation of geminiviral vector (pBY2eK) construct harboring SARS-CoV-2 RBD-Fc which was used for plant agroinfiltration. T-DNA region between LB (left border) and RB (right border) of pBY2eK consists of PinII 3′ (terminator from potato proteinase inhibitor II gene), P19 [P19 gene from tomato bushy stunt virus (TBSV)], TMVΩ 5'-UTR (5' untranslated region of tobacco mosaic virus Ω), P35S (cauliflower mosaic virus (CaMV) 35S promoter), LIR (long intergenic region of BeYDV genome), NbPsaK2T 5'UTR (5′ untranslated region), GOI (gene of interest), Ext3′FL (3′ full length of the tobacco (*Nicotiana tabacum*) extension gene), Rb7 (tobacco RB7 promoter), SIR (short intergenic region of BeYDV genome), and C2/C1 (bean yellow dwarf virus (BeYDV) open reading frames C1 and C2 encoding for replication initiation protein (Rep) and RepA). **(B)** Purified plant-produced SARS-CoV-2 RBD-Fc was analyzed by SDS-PAGE. **(C)** Western blotting of purified RBD-Fc fusion protein probed with goat anti-human IgG-HRP, and **(D)** anti-SARS-CoV-2 RBD-conjugated HRP. Lane M: marker; Lane 1: non-reducing condition; Lane 2: reducing condition.

The RBD-Fc fusion gene was cloned in the plant expression vector (Figure 1A). *N*. *benthamiana* plants were infiltrated with *A*. *tumefaciens* strain carrying pBYR2e-RBD-Fc construct. Agroinfiltrated leaves were harvested 4 days post-infiltration and the protein was purified by protein A column chromatography. The analysis by SDS-PAGE and western blot revealed the presence of two major bands with an apparent mass of ~150 kDa and ~75 kDa in non-reducing and reducing conditions, respectively, corresponding to the expected size of the protein (Figure 1B). The western blot analysis confirmed similar size bands when probed anti–human IgG-conjugated HRP (Figure 1C), and anti–RBD-conjugated HRP (Figure 1D) under reducing and non-reducing conditions.

### Baiya SARS-CoV-2 Vax 1 vaccine induces antibody response in monkeys

**Figure 2:**
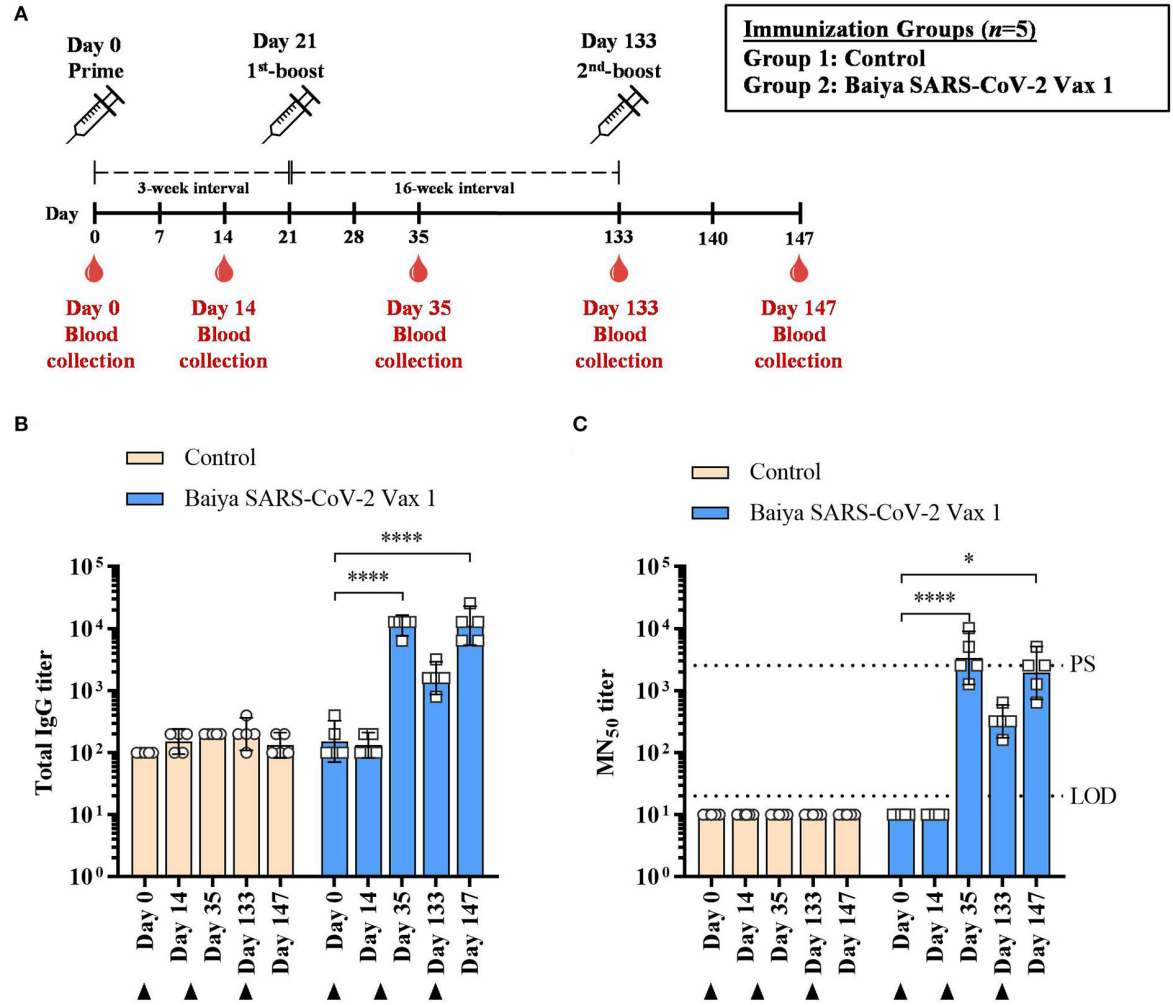
**(A)** Immunization and blood collection schedule of Baiya SARS-CoV-2 Vax 1 in cynomolgus monkeys. Monkeys were divided into two groups (*n* = 5), i.e., Baiya SARS-CoV-2 Vax 1 and control groups. Monkeys were immunized with a three-dose regimen (days 0, 21, and 133) and were bled on days 14, 35, 133, and 147. Response of **(B)** SARS-CoV-2 RBD-specific IgG titers and **(C)** 50% microneutralizing (MN_50_) titers in immunized animals. The animals were immunized with Baiya SARS-CoV-2 Vax 1 or alum only (control), on days 0, 21, and 133 (indicated by black arrows). Values smaller than the limit of detection (LOD) are plotted as 0.5*LOD. PS: positive serum. Data presented as GMT ± 95% CI of the endpoint titer in each group, *n* = 5. Two-way ANOVA, Dunnett's test was used (*p < 0.05, ****p < 0.0001).

The immunization schedule of Baiya SARS-CoV-2 Vax 1 is shown in Figure 2A. Baiya SARS-CoV-2 Vax 1 vaccine (10 μg) induced a robust IgG response 14 days after the second immunization on day 35 (GMT = 11,143) and the third immunization on day 147 (GMT = 11,143) which were significantly higher than the antibody titer estimated on day 0 (GMT = 152; *p* < 0.0001) (Figure 2B). As expected, 4-month lapse after the first boost (day 133), the IgG response significantly declined (GMT = 1,600). However, the second boost enhances the IgG response to be comparable with the IgG level after the first boost.

### Baiya SARS-CoV-2 Vax 1 vaccine induces neutralizing antibodies after immunization

Baiya SARS-CoV-2 Vax 1 vaccine induced neutralizing antibodies at 14 days after second immunization on day 35, (GMT = 3,378) and the third immunization on day 147 (GMT = 1,940) which were significantly higher than the pre-immunized sera collected on day 0 (GMT = 10) with *p* < 0.0001 and *p* < 0.05, respectively (Figure 2C). Noted that the levels of neutralizing antibodies on day 35 were higher than the positive serum (titer = 2,560), while a slightly lower titer was observed on the sera collected on day 147.

### Three-dose of Baiya SARS-CoV-2 Vax 1 vaccine can induce cross-reactive neutralizing antibodies against VOCs

**Figure 3:**
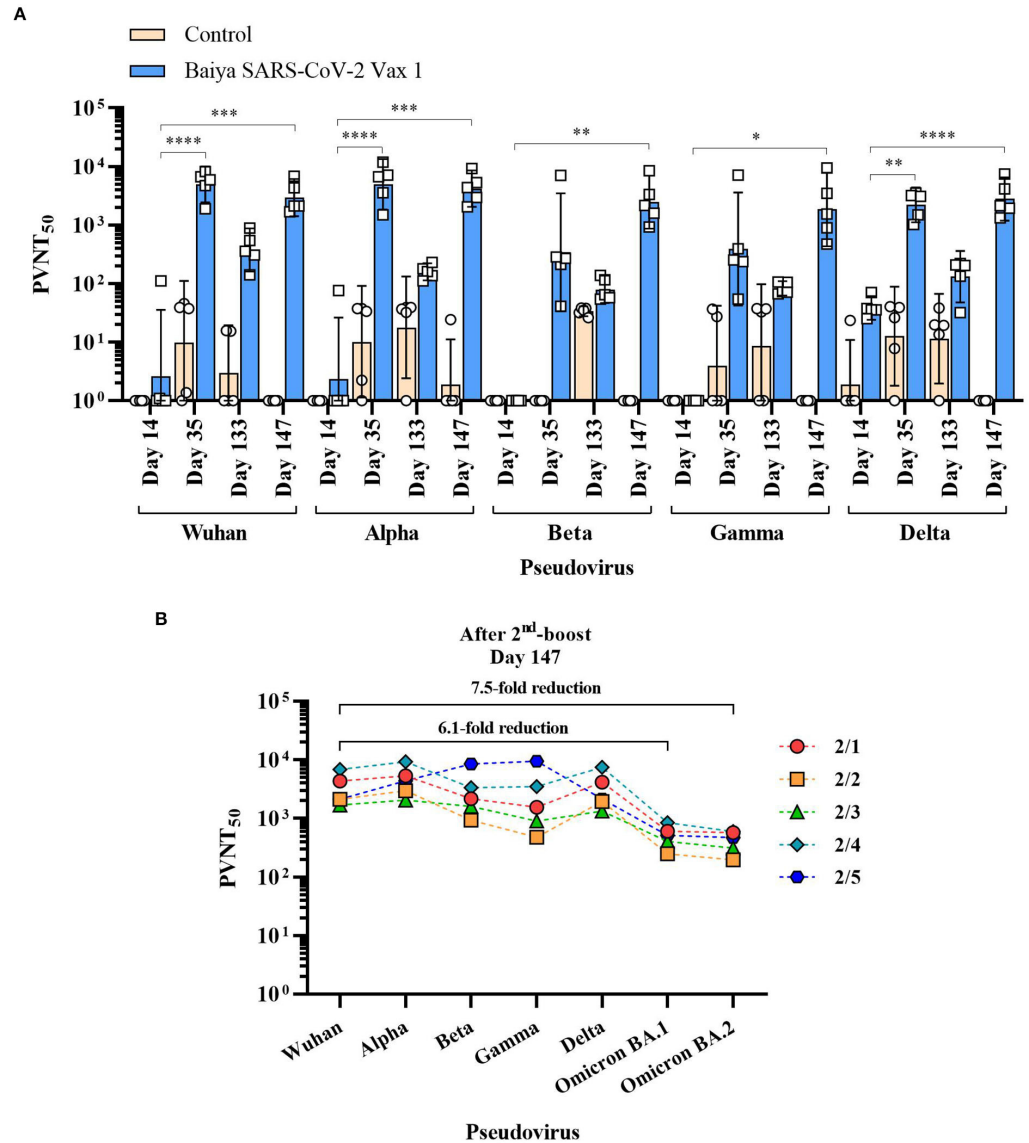
50% pseudovirus neutralization titers (PVNT_50_) of the immunized animal sera against SARS-CoV-2 and its variants. Animals were immunized with Baiya SARS-CoV-2 Vax 1 or alum only (control), on days 0, 21, and 133. **(A)** PVNT_50_ of immunized sera. Data presented as GMT ± 95% CI of the endpoint titer in each group, *n* = 5. Two-way ANOVA, Dunnett's test was used (*p < 0.05, **p < 0.01, ***p < 0.001, ****p < 0.0001). **(B)** Changes in PVNT_50_ of Baiya SARS-CoV-2 Vax 1 vaccinated individual animals against VOCs (2/1-2/5) on day 147 with numbers of fold reduction of GMT against Omicron BA.1 and BA.2 variants compared with Wuhan strain, *n* = 5.

The neutralizing antibodies induced by Baiya SARS-CoV-2 Vax 1 vaccination against Alpha, Beta, Gamma, and Delta VOCs were evaluated by spike pseudoviruses neutralization assay. The detectable neutralizing antibodies against all SARS-CoV-2 variants were observed after the second immunization on day 35. However, the neutralizing titers (PVNT_50_) for Wuhan and four VOCs significantly declined during the 4-month lapse (or day 133) after the second immunization (Figure 3A). The levels were either slightly higher or lower after the third immunization on day 147 as follows.

For Wuhan and Alpha variants, the PVNT_50_ on day 147 (GMT = 2,953 for Wuhan, and 4,197 for Alpha) was slightly lower than on day 35 (GMT = 4,937 for Wuhan, and 4,965 for Alpha). However, titers of sera collected on days 35 and 147 were significantly higher than on day 14 (GMT = 3 for Wuhan, *p* < 0.0001; and GMT = 2 for Alpha, *p* < 0.001), but it was not significantly different when compared with the sera on day 133 (GMT = 376 for Wuhan, and 159 for Alpha). For Beta and Gamma variants, the neutralizing activities of collected sera on day 147 (GMT = 2,464 for Beta, and 1,852 for Gamma) were not significantly higher than on day 35 (GMT = 343 or Beta, and 392 for Gamma) and day 133 (GMT = 80 for Beta, and 83 for Gamma), but it showed a significant increase when compared with sera tested on day 14 (GMT = 1 for Beta, *p* < 0.01; and GMT = 1 for Gamma, *p* < 0.05). For Delta variant, the neutralizing activity in sera on day 35 (GMT = 2,217, *p* < 0.01) and day 147 (GMT = 2,805, *p* < 0.0001) showed a significant increase when compared with the sera on day 14 (GMT = 38), but it was not significantly different when compared with the sera collected on day 133 (GMT = 132). The neutralizing activity against Omicron BA.1 (GMT = 484) and BA.2 strains (GMT = 396) on day 147 decreased when compared with other VOCs (Figure 3B). Compared to Wuhan strain, Omicron BA.1 and BA.2 showed 6.1- and 7.5-fold reductions in titers, respectively.

## Discussion

The COVID-19 pandemic has made a drastic negative impact on human health and the nation's economy worldwide. Thus far, hundreds of millions of people have been infected; hence detailed action plan along with effective vaccination is the key strategy to contain the virus spread and control the pandemic. Only a few vaccines are currently available for human use, and mass immunization may help attain herd immunity against the virus (Frederiksen et al., 2020; Malla et al., 2020). In addition, the recent emergence of SARS-CoV-2 variants /COVID-19 raises concern over the protective efficacy of existing vaccines against the infection caused by these variants. The mutations reported in the viral spike protein of SARS-CoV-2 variants confer resistance to neutralizing antibodies induced upon infection or vaccination (Hoffmann et al., 2021; Wang et al., 2021). Researchers worldwide are racing toward the development of effective COVID-19 vaccines with those against variants of concern being a global priority (Abdool Karim and De Oliveira, 2021).

For subunit vaccines, several expression systems were used, including bacteria, yeast, mammalian or plant cells (Hartwig et al., 2010; Tebianian et al., 2011; He et al., 2014; Chen et al., 2019). The advantages of the plant expression system for producing vaccine antigens in response to viral pandemics or epidemics have been well described (Hefferon, 2019; He et al., 2021). Our previous study indicated that 25 and 50 μg of Baiya SARS-CoV-2 Vax 1 could induce high levels of antibody titer. However, to increase the vaccine availability and accessibility during the pandemic, we thus reduced the dose of Baiya SARS-CoV-2 Vax 1 to 10 μg and tested the immunogenicity in cynomolgus monkeys. Alum was used as an adjuvant to enhance the immunogenicity of the subunit vaccine in this study as it holds excellent safety/efficacy profiles for long years and widely used in many available licensed vaccines, including tandem-repeat dimeric RBD subunit vaccine (Yang et al., 2021b). Alum salts also form a short-term depot at the injection site, eventually releasing the adsorbed antigen slowly to the body's immune response system (Gupta et al., 1993). The 10 μg Baiya SARS-CoV-2 Vax 1 induced neutralizing antibody responses at comparable levels to those of 25 and 50 μg Baiya SARS-CoV-2 Vax 1 (Siriwattananon et al., 2021). Nevertheless, this study demonstrated that RBD-specific IgG and neutralizing antibodies dramatically reduced after the 4-month lapse (day 133) after the second dose injection on day 21. Based on a previous study, the second dose of the RBD subunit vaccine for SARS-CoV induced a high humoral response in immunized mice, which dropped 3 months later (Du et al., 2007). Here, we showed that both RBD-specific IgG and neutralizing antibody titers against the Wuhan variant were at comparable levels between the second immunization (day 35) and the third immunization (day 147). This result was similar to the previous study in which the antibody response was shown to be increased after the booster dose (Du et al., 2007; Yang et al., 2021a).

As VOCs of SARS-CoV-2 spread worldwide, the vaccines' efficacy is a major concern (Bian et al., 2021; Lustig et al., 2021). Hence an effective vaccine against a broader range of SARS-CoV-2 variants is essential. Our data showed that Baiya SARS-CoV-2 Vax 1 vaccination elicits broadly neutralizing antibodies against VOCs. The neutralizing activity against SARS-CoV-2 variants was detectable within 14 days after two doses of immunization. Comparatively, the neutralization efficiency of the sera collected 4-month lapse (day 133) after the second dose injection was markedly reduced against Wuhan and variants. In contrast, the third vaccine dose substantially enhanced the neutralizing response against these strains, including the highly transmissible Delta strain. Although the neutralizing activity of the sera against variants was similar to the Wuhan strain, there is a slight reduction in the GMT titer against the Beta and Delta strain compared to the Wuhan strain even after the third immunization. Earlier reports showed the resistance of circulating variants against the antibody elicited after two doses of immunization (Cao et al., 2021; Chen et al., 2021; Wang et al., 2021; Zhou et al., 2021). Hence, the third dose of protein subunit vaccine may be ideal for boosting the immune response and providing protection against variants (Cao et al., 2021). However, the durability of neutralizing antibody responses needs to be investigated further.

The limitation of this study is that the pseudovirus displaying the full-length spike protein of SARS-CoV-2 variants was used instead of the authentic virus to test the neutralization potency of the immunized sera. However, this methodology is widely accepted as it provides results highly correlated with the neutralization of the infectious virus. Besides, this assay can be applied even in the absence of specific VOCs in the region, and the facility required is not restricted to biosafety level 3 (Schmidt et al., 2020; Shen et al., 2021a,b).

Further challenge studies are essential to assess whether the immunity induced by Baiya SARS-CoV-2 Vax 1 can protect animals against viral infection. Taken together, this study provides clear evidence that the Baiya SARS-CoV-2 Vax 1 is immunogenic and can elicit a neutralizing antibody response against SARS-CoV-2 variants in monkeys. However, toxicity and efficacy studies are also vital before clinical applications.

In conclusion, our data highlight the potential of Baiya SARS-CoV-2 Vax 1 in inducing a significant humoral immune response in cynomolgus macaques with a three-dose vaccination regime. In addition, the sera from the immunized monkeys were found to be effective in neutralizing SARS-CoV-2 variants recognized by the WHO as variants of concern. These results open an avenue for using the plant-produced protein subunit vaccine in the fight against SARS-CoV-2. In addition, a three-dose regime can be suggested for the countries where the infection is severe. Further, a three-dose vaccination regime can be employed for developing an effective vaccine against SARS-CoV-2 and its variants. However, the durability immune response needs to be investigated.

## Data availability statement

The original contributions presented in the study are included in the article/supplementary material, further inquiries can be directed to the corresponding author.

## Ethics statement

The animal study was reviewed and approved by the National Primate Research Center of Thailand-Chulalongkorn University (NPRCT-CU) Animal Care and Use Committee, Chulalongkorn University, Thailand.

## Author contributions

AT, AJ, and WP designed all experiments. NK, BS, KR, and CP performed protein expression, protein purification, and ELISA. TK, NS, and SMal performed vaccination in non-human primates. SMan and AT performed a microneutralization assay. KS and AJ performed a pseudovirus neutralization assay. NK and BS drafted the manuscript. All authors analyzed the data, revised the manuscript, and approved it for publication.

## Funding

The authors declare that this study received funding from Baiya Phytopharm Co., Ltd.. The funder was not involved in the study design, collection, analysis, interpretation of data, the writing of this article, or the decision to submit it for publication.

## Conflict of interest

Author WP from Chulalongkorn University is a founder/shareholder of Baiya Phytopharm Co., Ltd. Thailand. Authors BS and KR were employed by Baiya Phytopharm Co., Ltd. The remaining authors declare that the research was conducted in the absence of any commercial or financial relationships that could be construed as a potential conflict of interest.

## Publisher's note

All claims expressed in this article are solely those of the authors and do not necessarily represent those of their affiliated organizations, or those of the publisher, the editors and the reviewers. Any product that may be evaluated in this article, or claim that may be made by its manufacturer, is not guaranteed or endorsed by the publisher.

## Abbreviations

A450: Absorbance at 450 nm
Al content: Aluminum content
Alum: Aluminum hydroxide
BSL: Biosafety level
CI: Confidence interval
COVID-19: Coronavirus disease 2019
DMEM: Dulbecco's modified eagle medium
Fc region: Fragment crystallizable region
GMT: Geometric mean titer
hACE2: Human angiotensin-converting enzyme 2
HRP: Horseradish peroxidase
IgG: Immunoglobulin G
LOD: Limit of detection
MERS-CoV: Middle East respiratory syndrome coronavirus
MN50 titer: 50% microneutralizing titer
N: Nucleocapsid glycoprotein
NHP: Non-human primate
NPRCT-CU: National Primate Research Center of Thailand-Chulalongkorn University
PBS: Phosphate-buffered saline
PBST: 1xPhosphate-buffered saline with 0.05% Tween 20
PS: Positive serum
PVNT50: 50% pseudovirus neutralizing titer
RBD: Receptor-binding domain
RT: Room temperature
S: Spike or surface glycoprotein
SARS-CoV: Severe acute respiratory syndrome coronavirus
SARS-CoV-2: Severe acute respiratory syndrome coronavirus 2
TCID50: 50% tissue culture infective dose
TMB: Tetramethylbenzidine
VOC: Variant of concern
WHO: World Health Organization.
